# The relationship between fat distribution and diabetes in US adults by race/ethnicity

**DOI:** 10.3389/fpubh.2024.1373544

**Published:** 2024-02-20

**Authors:** Furong Xu, Jacob E. Earp, Deborah Riebe, Matthew J. Delmonico, Ingrid E. Lofgren, Geoffrey W. Greene

**Affiliations:** ^1^College of Education, University of Rhode Island, Kingston, RI, United States; ^2^Department of Kinesiology, University of Connecticut, Storrs, CT, United States; ^3^Department of Kinesiology, University of Rhode Island, Kingston, RI, United States; ^4^Department of Nutrition, University of Rhode Island, Kingston, RI, United States

**Keywords:** diabetes, type 2 diabetes, visceral fat, subcutaneous fat area, adults

## Abstract

**Introduction:**

This study examined the relationship between fat distribution and diabetes by sex-specific racial/ethnic groups.

**Methods:**

A secondary data analysis of National Health and Nutrition Examination Survey 2011–2018 data (*n* = 11,972) was completed. Key variables examined were visceral adipose tissue area (VATA), subcutaneous fat area (SFA), diabetes prevalence, and race/ethnicity. The association of VATA and SFA and diabetes prevalence was examined separately and simultaneously using multiple logistic regression. Bonferroni corrections were applied to all multiple comparisons between racial/ethnic groups. All analyses were adjusted for demographics and muscle mass.

**Results:**

VATA was positively associated with diabetes in both sexes (*p* < 0.001) and across all racial/ethnic groups (*p* < 0.05) except Black females. No statistically significant relationships were observed between SFA and diabetes while accounting for VATA with the exception of White females (*p* = 0.032). When comparing racial/ethnic groups, the relationship between VATA and diabetes was stronger in White and Hispanic females than in Black females (*p* < 0.005) while the relationship between SFA and diabetes did not differ between any racial/ethnic groups.

**Conclusion:**

This study found that VATA is associated with diabetes for both sexes across almost all racial/ethnic groups independent of SFA whereas the only significant relationship between SFA and diabetes, independent of VATA, was observed in White females. The findings indicated that visceral fat was more strongly associated with diabetes than subcutaneous. Additionally, there are health disparities in sex-specific racial/ethnic groups thus further study is warranted.

## 1 Introduction

Out of 37.3 million Americans with diabetes, over 90% have type 2 diabetes (T2D) (1). T2D is a chronic disease resulting from decreased insulin sensitivity and is strongly related to excessive body weight (2, 3). In the past two decades, an abundance of research has indicated that regional fat distribution is a greater risk factor for T2D than overall body fat (4–16) with visceral adipose tissue exhibiting the strongest relationship (4–14). However, studies regarding visceral adipose tissue and T2D have been limited to either a single race Caucasians (4, 5) or primarily Caucasians (6), Asian populations (7, 8), physician recruited patients (9), or did not have a direct measure of visceral adipose tissue (10, 11). Visceral adipose tissue area (VATA) is often used in research as a measure of visceral adiposity (7, 8). In contrast to VATA, the relationship between subcutaneous adipose and T2D is less clear; with studies reporting both positive (9) and negative associations between subcutaneous fat and T2D (6). Studies examining the relationship between subcutaneous fat and insulin resistance are also equivocal, with studies reporting either no association (12, 13) or a positive association (14–17). Furthermore, although one study compared the relationships both of VATA and subcutaneous fat and T2D in racial/ethnic groups (9), their study sample was limited to physician (e.g., primary care, cardiologists, diabetologists, endocrinologists) referrals (9). Thus, the effects of race/ethnicity on these relationships needs additional research in the general population, especially in a representative adult population, given previous observations that race/ethnicity affect both regional fat distribution and T2D prevalence (18). Further studies on the relationship of both VATA and subcutaneous adipose tissue with T2D using direct measures are warranted using a nationally representative sample. Accordingly, the aims of the present study were to use a nationally representative sample of the US adult population with direct measures of body composition to (1) describe the relationship between VATA and subcutaneous adipose and diabetes by sex and race/ethnicity, (2) examine whether there is a stronger relationship between diabetes and VATA or subcutaneous fat.

## 2 Method

This is a cross-sectional study utilizing National Health and Nutrition Examination Survey (NHANES) 2011–2018 data (*n* = 39,156) (19, 20). For this study, participants were included if they (1) were adults aged 20 or older and eligible for the dual-energy X-ray absorptiometry (DXA) test (*n* = 14,934), (2) had DXA data (*n* = 11,972), (3) had both self-reported diabetes data and measured blood glucose and/or glycohemoglobin data (*n* = 11,972), (4) had measured height, and weight (*n* = 11,972). A total of 11,972 adults fulfilled our study criteria and were included in the final analysis. This study was approved by the University of Rhode Island’s Institutional Review Board (IRB, #1991682-2).

### 2.1 Fat distribution

Two fat distribution indices were used in this analysis including: VATA (cm^2^) and subcutaneous fat area (SFA) (cm^2^). VATA and SFA were derived from the DXA output from the NHANES dataset (21).

### 2.2 Diabetes

Respondents were classified as having diabetes if they met at least one of the following criteria. The first was diagnosed diabetes based on self-report; a positive response to the question “Have you been told by a doctor or health professional that you have diabetes other than during pregnancy?” The second was if respondents responded “yes” to either or both of the following questions: (1) “Are you now taking insulin?,” (2) “Are you now taking diabetic pills to lower blood sugar?” (22, 23). The third was a measured glycohemoglobin ≥6.5% or fasting glucose ≥126 mg/dL, which are diagnostic criteria outlined in the Center for Disease Control’s definition for undiagnosed diabetes (23).

### 2.3 Demographics

The following demographic variables were included in the analysis: (1) Age (yrs), (2) Self-reported race/ethnicity (classifications were White, Black, Hispanic, Asian, or Others which included multi-ethnicity), (3) Education (high school or less, some college or more), and (4) Poverty to income ratio (below <1, at or above ≥1 poverty level) (19, 20). Height (cm) and weight (kg) were also included and used to calculate body mass index (BMI) which was used to further classify respondent weight status – underweight (BMI < 18.5 kg/m^2^), normal (18.5 kg/m^2^ ≤ BMI ≤ 24.9 kg/m^2^), overweight (25.0 kg/m^2^ ≤ BMI ≤ 29.9 kg/m^2^), and obese (BMI ≥ 30 kg/m^2^) (21, 24).

### 2.4 Covariates

Four demographic variables (age, race/ethnicity, education, and family income) were included as covariates (19, 25). In addition, height was included as people often experience an increase in both muscle mass and fat mass with increasing height. However, these increases could be disproportional and can differentially affect T2D risk particularly as visceral adipose tissue is more inflammatory than subcutaneous adipose while muscle mass is anti-inflammatory (20, 26, 27). Therefore, muscle mass was also included as a covariate along with height and demographics. Muscle mass was derived from DXA output as total body lean mass excluding bone mineral content (21). Muscle mass has been found to have a positive influence on blood glucose regulation (28–31). Controlling for height and muscle mass as well as demographics should reduce variance that is unrelated to the relationship of fat distribution and T2D.

### 2.5 Data analysis

The combined eight-year sample weight was used in all analyses given the complexity of the sample (32). Demographic information and other participant characteristics were reported as weighted mean ± standard errors or count (weighted percentages) as appropriate, for both the total study population and male and female cohorts. To investigate the relationship between fat distribution and diabetes, descriptive statistics produced the distribution of VATA and SFA according to diabetes and non-diabetes, and the difference was tested using a *t*-test (PROC SURVEYREG in SAS). For the association of the VATA and SFA with diabetes, odds ratio (OR) and 95% confidence intervals (95% CI) were generated using multiple logistic regression. The initial models adjusted for age, race/ethnicity, education, family income, height and muscle mass. VATA and SFA were analyzed as continuous variables. Subgroup differences by race/ethnicity were analyzed separately via *post-hoc* analysis to compare the difference between any two race/ethnicities. A Bonferroni correction was applied for those multiple comparisons between racial/ethnic groups. Effect modification was assessed using the Wald test. To compare the effect of VATA and SFA on diabetes, multivariate analyses were performed in the final model. Both VATA and SFA were included in the final model to estimate standardized coefficient [parameter estimates multiplied by the standard deviation (SD) of the variable], adjusted for covariates of age, race/ethnicity, education, family income, height, and muscle mass. All analyses were performed using SAS Version 9.4 (SAS Institute, Cary, North Carolina, USA). A two-sided *p* < 0.05 was considered statistically significant. For multiple comparisons between racial/ethnic groups *p* < 0.005 was considered statistically significant as the result of the Bonferroni correction (desired alpha level divided by 10 racial/ethnic group comparisons).

## 3 Results

### 3.1 Respondent characteristics

**Figure 1:**
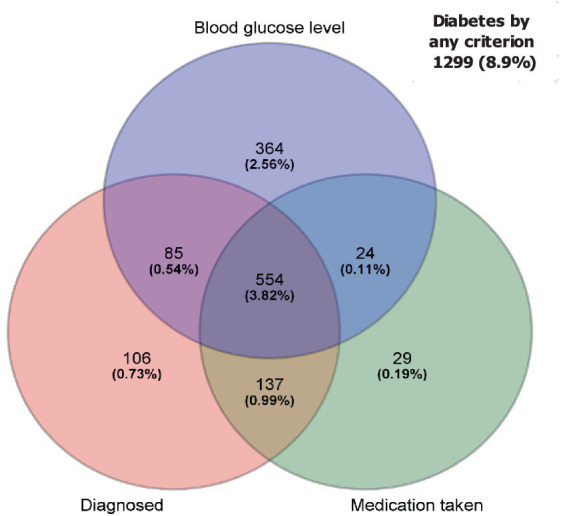
Venn diagram of overlap for diabetes among diagnosed, medication taken, and blood glucose level. Out of 11,972 respondents, 418 was not included in this figure due to missing blood test. As the result of that, 11,554 were used for this figure.

Approximately half of the 11,972 respondents were females (48.4%), 38.4% were non-White minorities, 34.7% had high school or less education background, 15.8% lived below the poverty level, 31.9% and 37.6% with overweight and obesity, respectively. In total, 6.0% reported diagnosed diabetes, 5.1% were taking medication either diabetic pills or insulin, and 7.0% were found to have undiagnosed diabetes according to blood glucose or glycohemoglobin (see Table 1 and Figure 1). Sex and race/ethnicity-specific fat distribution in Table 2 indicated that respondents with diabetes had higher VATA and SFA in comparison to respondents without diabetes regardless of sex and racial/ethnic groups (see Table 2).

**Table 1:** Demographic characteristics, NHANES 2011–2018.

Variables	Total	Male	Female	Value of *p*
	*n* = 11,972	*n* = 6,065 (51.6%)	*n* = 5,907 (48.4%)	
Age (yrs)	39.69 ± 0.23	39.32 ± 0.22	40.08 ± 0.30	0.005*
Race/ethnicity, n (%)				
White	4,166 (61.6)	2,147 (61.5)	2,019 (61.8)	0.771
Black	2,676 (11.8)	1,325 (11.3)	1,351 (12.4)	0.009*
Hispanic	2,911 (17.0)	1,423 (17.7)	1,488 (16.3)	0.003*
Asian	1,699 (5.8)	884 (5.6)	815 (5.9)	0.186
Others	520 (3.8)	286 (3.9)	234 (3.6)	0.592
Education, n (%)				
High school or less	4,806 (34.7)	2,683 (38.7)	2,123 (30.4)	<0.001*
Some college or more	7,164 (65.3)	3,382 (61.3)	3,782 (69.6)	<0.001*
Poverty income ratio, n (%)				
<1.0	2,500 (15.8)	1,189 (14.6)	1,311 (17.1)	<0.001*
≥1.0	8,484 (84.2)	4,355 (85.4)	4,129 (82.9)	<0.001*
Height (cm)	83.60 ± 0.39	89.49 ± 0.46	77.33 ± 0.48	<0.001*
Weight (kg)	169.54 ± 0.15	176.03 ± 0.17	162.62 ± 0.17	<0.001*
Body Mass Index (kg/m^2^)	29.01 ± 0.13	28.81 ± 0.14	29.23 ± 0.18	0.029*
Underweight n (%)	243 (1.7)	109 (1.4)	134 (2.1)	0.012*
Normal n (%)	3,476 (28.8)	1,692 (25.7)	1,784 (32.2)	<0.001*
Overweight n (%)	3,721 (31.9)	2,169 (36.4)	1,552 (27.1)	<0.001*
Obese n (%)	4,532 (37.6)	2,095 (36.5)	2,437 (38.7)	0.106
Total Diabetes, n (%)	1,319 (8.8)	683 (9.3)	636 (8.3)	0.208
Diagnosed diabetes	899 (6.0)	459 (6.2)	440 (5.9)	0.549
Medication taken	758 (5.1)	386 (5.3)	372 (4.9)	0.415
Blood glucose level	1,029 (7.0)	564 (8.0)	465 (6.1)	0.003*
Glycohemoglobin (%)	5.53 ± 0.01	5.55 ± 0.02	5.50 ± 0.01	0.009*
Fasting glucose (mg/dL)	104.51 ± 0.50	107.00 ± 0.73	101.80 ± 0.55	<0.001*
VATA (cm^2^)	105.66 ± 1.19	112.77 ± 1.35	98.08 ± 1.47	<0.001*
SFA (cm^2^)	340.76 ± 3.23	280.17 ± 3.29	405.38 ± 4.26	<0.001*
Muscle mass (g)	52566.37 ± 198.43	60920.84 ± 238.28	44027.54 ± 193.35	<0.001*

Data are presented as weighted mean ± standard errors unless otherwise specified; value of *p* was obtained by performing *t*-test (PROC SURVEYREG in SAS) for continuous. NHANES, National Health and Nutrition Examination Survey; VATA, visceral adipose tissue area; SFA, Subcutaneous fat area. *p <0.05.

**Table 2:** The fat distribution by sex and diabetes status, NHANES 2011–2018.

	Male			Female		
	Diabetes	Non-diabetes	Value of *p*	Diabetes	Non-diabetes	Value of *p*
Overall	*n* = 683	*n* = 5,382		*n* = 636	*n* = 5,271	
VATA (cm^2^)	166.73 ± 3.62	107.25 ± 1.29	<0.001*	162.62 ± 3.66	92.22 ± 1.38	<0.001*
SFA (cm^2^)	358.30 ± 9.09	272.18 ± 3.21	<0.001*	523.54 ± 8.88	394.64 ± 4.17	<0.001*
White						
VATA (cm^2^)	183.91 ± 5.31	112.95 ± 1.81	<0.001*	180.85 ± 7.18	92.91 ± 1.64	<0.001*
SFA (cm^2^)	375.32 ± 15.52	275.46 ± 4.16	<0.001*	528.26 ± 16.17	389.09 ± 5.15	<0.001*
Black						
VATA (cm^2^)	132.17 ± 4.83	78.98 ± 1.20	<0.001*	124.24 ± 3.35	84.09 ± 1.65	<0.001*
SFA (cm^2^)	368.73 ± 12.12	242.88 ± 5.56	<0.001*	578.20 ± 10.65	452.99 ± 6.31	<0.001*
Hispanic						
VATA (cm^2^)	160.56 ± 4.25	110.56 ± 1.71	<0.001*	167.94 ± 3.80	102.02 ± 1.95	<0.001*
SFA (cm^2^)	340.65 ± 9.38	289.77 ± 5.32	<0.001*	499.05 ± 10.93	408.11 ± 5.29	<0.001*
Asian						
VATA (cm^2^)	133.85 ± 3.82	90.30 ± 1.62	<0.001*	120.73 ± 4.88	72.80 ± 1.41	<0.001*
SFA (cm^2^)	262.09 ± 9.54	222.28 ± 3.90	<0.001*	371.43 ± 14.63	296.61 ± 3.24	<0.001*
Others						
VATA (cm^2^)	154.85 ± 9.32	104.09 ± 4.12	<0.001*	148.59 ± 11.85	95.57 ± 6.09	<0.001*
SFA (cm^2^)	339.35 ± 25.16	294.74 ± 13.77	0.128	594.30 ± 37.16	401.37 ± 10.94	<0.001*

*P*-value was obtained by performing *t*-test (PROC SURVEYREG in SAS) for continuous variables, and Chisq-test (PROC SURVEYFREQ in SAS) for categorical variables; VATA, visceral adipose tissue area; SFA, Subcutaneous fat area. *p <0.05.

### 3.2 Initial model analysis

Adjusted analytical results for the relationship of VATA and SFA with diabetes are reported in Table 3. The results indicated that fat distribution was associated with diabetes. More specifically, for every SD of VATA increase, the odds of having diabetes increased 78% in males (OR = 1.78, 95% CI: 1.52–2.08) and 100% in females (OR = 2.00, 95%CI:1.67, 2.41). Males were 1.41 times more likely to have diabetes for every SD increase in SFA, but the relationship between SFA and diabetes in females was not statistically significant (see Table 3).

**Table 3:** The association between VATA and SFA with diabetes by race/ethnicity among US adults (reduced model).

	Total (*n* = 11,972)		White (*n* = 4,166)		Black (*n* = 2,676)		Hispanic (*n* = 2,911)		Asian (1,699)		Others (*n* = 520)		Interaction value of *p*
	Adj. OR (95% CI)	*p*-value	Adj. OR (95% CI)	*p*-value	Adj. OR (95% CI)	*p*-value	Adj. OR (95% CI)	*p*-value	Adj. OR (95% CI)	*p*-value	Adj. OR (95% CI)	*p*-value	
Male	*n* = 6,065		*n* = 2,147		*n* = 1,325		*n* = 1,423		*n* = 884		*n* = 286		
VATA – per SD increase	1.78 (1.52, 2.08)	<0.001*	1.77 (1.43, 2.19)	<0.001*	1.95 (1.41, 2.71)	<0.001*	1.62 (1.15, 2.28)	0.004*	2.49 (1.63, 3.81)	<0.001*	1.71 (0.89, 3.30)	0.099	0.202
SFA – per SD increase	1.41 (1.18, 1.68)	<0.001*	1.53 (1.12, 2.09)	0.006*	1.56 (1.26, 1.92)	<0.001*	1.25 (0.91, 1.71)	0.157	1.80 (1.14, 2.84)	0.01*	0.79 (0.38, 1.61)	0.498	0.044*
Female	*n* = 5,907		*n* = 2,019		*n* = 1,351		*n* = 1,488		*n* = 815		*n* = 234		
VATA – per SD increase	2.00 (1.67, 2.41)	<0.001*	2.29 (1.68, 3.12)^a^	<0.001*	1.23 (1.00, 1.51)	0.049*	1.82 (1.43, 2.32)^b^	<0.001*	2.90 (1.84, 4.58)^c^	<0.001*	1.30 (0.50, 3.38)^d^	0.582	<0.001*
SFA – per SD increase	0.97 (0.77, 1.22)	0.787	0.86 (0.60, 1.22)	0.386	1.14 (0.84, 1.56)	0.391	1.08 (0.78, 1.51)	0.634	1.81 (1.00, 3.29)^c^	0.046*	0.56 (0.15, 2.09)^d^	0.387	0.011*

For the reduced model, adjusted odds ratios (ORs) were obtained by multiple logistic regression, adjusted for age, education, family income, height and muscle mass. Interaction *p*-values were estimated to access the modification effect of race/ethnicity on the association between fat distribution and diabetes. *Post-hoc* analysis to compare the difference between any two race/ethnicity, which Bonferroni correction applied [divide the critical value of *p* by 10 comparisons being made (0.05/10 = 0.005)]. ^a^*p* < 0.005 by comparing White vs. Black, ^b^*p* < 0.005 by comparing Black vs. Hispanic, ^c^*p* < 0.005 by comparing Black vs. Asian, ^d^*p* < 0.005 by comparing Asian vs. Others, SD = standard deviation. VATA, visceral adipose tissue area; SFA, Subcutaneous fat area; VSR, visceral to subcutaneous fat ratio. *p < 0.05.

Table 3 shows the relationships of fat distribution with diabetes by race/ethnicity, comparisons of those relationships across racial/ethnic groups, and the interaction value of *p* for effect modification of race/ethnicity on such relationships. In males, for every SD of VATA increase, the odds of having diabetes increased 1.77, 1.95, 1.62, and 2.49 times for White, Black, Hispanic, and Asian adults respectively, but the interaction was not statistically significant and the relationship between VATA and diabetes did not differ by racial/ethnic groups. For each SD increase in SFA, the odds of having diabetes increased 1.53, 1.56, and 1.80 times for White, Black and Asian, respectively. However, the relationship differences between SFA and diabetes was not observed between racial/ethnic groups in males after the Bonferroni correction (*p* > 0.005). For females, VATA was positively associated with diabetes in White (OR = 2.29, 95%CI: 1.68, 3.12), Black (OR = 1.23, 95%CI: 1.00, 1.51), Hispanic (OR = 1.82, 95%CI: 1.43, 2.32) and Asian (OR = 2.90, 95%CI: 1.84, 4.58). The interaction was significant (*p* < 0.001), and the relationship between VATA and diabetes was stronger in White, Hispanic, and Asian females in comparison to Black females. The relationship between SFA and diabetes was only statistically significant in Asian females, and the relationship between SFA and diabetes was stronger in Asian females than in Black females (see Table 3).

### 3.3 The full model analysis

Table 4 compares the effect of VATA and SFA on diabetes adjusted by age, race/ethnicity, education, family income, height, and muscle mass. Adjusted standard estimates were conducted to compare VATA and SFA in the odds of getting diabetes per SD increase by sex and race/ethnicity. Regardless of sex and racial/ethnical groups, when VATA was controlled for SFA there was little effect modification. Specifically, for males, each SD increase in VATA, on average, increased the odds of having diabetes by 75% (*p* < 0.001). For females, the increase was 107% per one SD increase in VATA (*p* < 0.001). However, when SFA was controlled for VATA, there was no effect in both sexes (see Table 4). In addition, although the race/ethnicity specific analysis (Table 4) revealed that the relationship between VATA and diabetes remains stronger than the relationship between SFA and diabetes, there were no differences between racial/ethnic groups in males. In females, the relationship between VATA and diabetes was stronger in both White (OR = 2.46, 95%CI: 1.75, 3.46) and Hispanic (OR = 1.86, 95% CI: 1.43, 2.42) females than Black (OR = 1.21, 95%CI: 0.98, 1.51) females (*p* < 0.005) with the Bonferroni correction. There was an inverse relationship observed between SFA and diabetes in White females (*p* = 0.032) but the results for SFA were non-significant in other racial/ethnic groups for females (Table 4).

**Table 4:** The association between VATA and SFA with diabetes by race/ethnicity among US adults (full model).

	Total (*n* = 11,972)		White (*n* = 4,166)		Black (*n* = 2,676)		Hispanic (*n* = 2,911)		Asian (1,699)		Others (*n* = 520)		Interaction *p*-value
	Adj. OR (95% CI)	*p*-value	Adj. OR (95% CI)	*p*-value	Adj. OR (95% CI)	*p*-value	Adj. OR (95% CI)	*p*-value			Adj. OR (95% CI)	*p*-value	
Male	*n* = 6,065		*n* = 2,147		*n* = 1,325		*n* = 1,423		*n* = 884		*n* = 286		
VATA – per SD increase	1.75 (1.47, 2.08)	<0.001*	1.72 (1.36, 2.16)	<0.001*	1.77 (1.22, 2.58)	0.002*	1.63 (1.11, 2.38)	0.01*	2.57 (1.53, 4.31)	<0.001*	2.28 (1.24, 4.22)	0.007*	0.343
SFA – per SD increase	1.04 (0.84, 1.29)	0.707	1.09 (0.75, 1.59)	0.628	1.27 (0.97, 1.64)	0.07	0.99 (0.67, 1.47)	0.972	0.93 (0.48, 1.80)	0.822	0.51 (0.22, 1.21)	0.118	0.177
Female	*n* = 5,907		*n* = 2,019		*n* = 1,351		*n* = 1,488		*n* = 815		*n* = 234		
VATA – per SD increase	2.07 (1.70, 2.51)	<0.001*	2.46 (1.75, 3.46)	<0.001*	1.21 (0.98, 1.51)^a^	0.073	1.86 (1.43, 2.42)^b^	<0.001*	2.80 (1.64, 4.77)	<0.001*	1.40 (0.58, 3.37)	0.44	0.003*
SFA – per SD increase	0.80 (0.61, 1.04)	0.084	0.62 (0.40, 0.97)	0.032*	1.12 (0.81, 1.54)	0.495	0.89 (0.59, 1.35)	0.576	1.10 (0.49, 2.46)	0.817	0.51 (0.14, 1.83)	0.289	0.375

For the full model, both VATA and SFA were put into the full model, adjusted odds ratios (ORs) were obtained by multivariate analyses, adjusted for age, race/ethnicity, education, family income, height and muscle mass. Interaction *p*-values were estimated to access the modification effect of race/ethnicity on the association between fat distribution and diabetes. *Post-hoc* analysis to compare the difference between any two race/ethnicity, which Bonferroni correction applied [divide the critical *p* value by the number of comparisons being made (0.05/10 = 0.005)]. ^a^*p* < 0.005 by comparing White vs. Black, ^b^*p* < 0.005 by comparing Black vs. Hispanic. SD, standard deviation; VATA, visceral adipose tissue area; SFA, Subcutaneous fat area. *p < 0.05.

## 4 Discussion

The current study, using a nationally representative sample with direct measurement of VATA and SFA revealed that VATA was associated with diabetes in US adults independent of SFA. The relationship between VATA and diabetes was not affected by race/ethnicity in males, whereas there were differences in the relationship between VATA and diabetes for females among racial/ethnic groups. In contrast, these relationships between SFA and diabetes were not present in any racial/ethnic groups in males and females when VATA was accounted for in the analysis. The only exception was the inverse relationship observed between SFA and diabetes in White females. Although national statistics indicate that at least 90% of persons with diabetes have T2D (1), the type of diabetes was not differentiated in the current study. Therefore, we use diabetes instead of T2D in the discussion and regard this as one of our study limitations.

In the initial model, VATA was examined independently for its relationship with diabetes. These analyses found that VATA was positively associated with diabetes, which is a result that is consistent with previous studies (4–11). However, the present study expanded upon previous studies by utilizing nationally representative young and middle-aged US adults and by using direct measurement of VATA and SFA. Previous studies were limited to a single race/ethnicity (4–8), used regional samples (6) or estimated VATA rather than using a direct measure (10, 11). There were positive relationships between VATA and diabetes in all racial/ethnic groups in both sexes, however the strength of these relationships significantly differed between females of different race/ethnicity classifications. Specifically, the relationship between VATA and diabetes was stronger in White, Hispanic and Asian females compared to Black females. In contrast to the present results, Nazare et al. (9) failed to find an effect of race/ethnicity on the relationship between VATA and T2D. However, their study utilized a sample that was one-third of the size of the present study (1,955 vs. 5,828 women), therefore, it is possible that the previous study was unable to observe this potentially clinically significant effect due to the limited sample size. While the present study observed an effect of race/ethnicity on the relationship between VATA and diabetes, it’s beyond the scope of the present study to determine the cause of these discrepancies. However, it is possible the influence of VATA on insulin resistance varied among racial/ethnic groups in women. Therefore, further studies on this are warranted.

Previous studies on the relationship between SFA and diabetes have provided equivocal results (6, 9, 12, 13, 33). The present study adds to this topic by both utilizing a large representative sample of US adults and by investigating this relationship when analyzed with and without accounting for VATA. In the present study, there was a positive relationship between SFA and diabetes observed in all males, in White, Black, and Asian males, and Asian females without accounting for VATA. Unlike the present study, Borel and colleagues (12) found SFA had an inverse relationship with diabetes in women but no association in men. Other studies found SFA was positively associated with T2D in women only (33), or Hispanic men and women (9) but no racial/ethnic group difference (12). The conflicting findings could be due to sample differences such as Asian only (33) patients recruited by physicians (9, 12), or by the covariates used in the analysis. It is worth noting that the present study adjusted for muscle mass thus the outcome is independent of the muscle mass effect which has not been done in previous studies (6, 9, 12, 13, 33). We believe controlling for the effect of muscle mass in the analysis is necessary given its connection to blood glucose and T2D (28–31). The findings for SFA indicated SFA in White, Black and Asian males, and Asian females might be a diabetes risk factor to consider. Further studies on the racial/ethnic-specific SFA risk and T2D are warranted.

The secondary purpose of the present study was to explore the relationship between both VATA and SFA and diabetes when accounting for fat distribution (full model). Both in isolation and when accounting for SFA, VATA was positively related to diabetes in males and females, and in all racial ethnic groups except Black females. This agrees with previous research that reported a disassociation between VATA and insulin resistance in Black women of African descent (34). In contrast, while SFA was related to diabetes in men when analyzed in isolation, no statistically significant relationships between SFA and diabetes were observed in individual racial/ethnic groups when VATA was accounted for in any group. For females, SFA was inversely related to diabetes only in White females independent of VATA. Nevertheless, it can be concluded that VATA is independently associated with diabetes. Many studies have investigated the effect of VATA on diabetes (4–8, 10, 11, 33, 35–37), and some studies on SFA and diabetes (6, 9, 35–37), they are limited to (1) single race/ethnicity (4–8, 33), (2) estimation of fat distribution instead of direct measures (10, 11). Although some previous studies examined racial/ethnic specific relationship between fat distribution and diabetes, they were conducted among adults 60 years or older (35–37), who may differ in insulin resistance than adults less than 60 years of age (38). Most importantly, VATA and SFA was not independently examined even though previous research suggested that they have different deleterious impact on insulin sensitivity (17), and muscle mass was not adjusted despite its positive influence on blood glucose regulation (28–31). To the best of our knowledge, no research has examined the relationship of VATA and SFA and diabetes independent of SFA or VATA using a representative sample of young and middle-aged US adults. Given both VATA and SFA are positively associated with overall adiposity and body mass (39), these results contextualize VATA and SFA’s relationship with diabetes as possible risk factors but there is variation by sex and race/ethnicity. Further research in this area is warranted.

### 4.1 Study strengths and limitations

Strengths of the present study included (1) a large representative sample of US young and middle-aged adults, (2) use of DXA to measure VATA and SFA following standard lab procedures (19), (3) examination of the relationship of fat distribution and diabetes across racial/ethnic groups allowing a study of the effect modification of race/ethnicity on the relationship of fat distribution and diabetes, (4) adjustment for both height and muscle mass accounting for individual body size differences independent of muscle mass, and (5) Bonferroni correction applied to all multiple comparison between racial/ethnic groups to avoid false positive results. The limitations of the present study include (1) the NHANES dataset did not differentiate between type 1 diabetes and T2D although the Centers for Disease Control and Prevention‘s National Diabetes Statistics Report indicated that higher percentage of (90–95%) of diagnosed diabetes are T2D (1), and (2) concerns about the accuracy of self-report for diabetes diagnosis and for taking diabetes medications (1, 40).

## 5 Conclusion

Results demonstrate that visceral adipose tissue was associated with diabetes independent of subcutaneous adipose tissue in both males and females and all racial/ethnic groups except Black females. The magnitude of the association of visceral fat and diabetes was stronger in White and Hispanic females than in Black females. Whereas subcutaneous adipose tissue was associated with diabetes independent of visceral adipose tissue only in White Females. Visceral adipose tissue was more highly related to diabetes compared to subcutaneous adipose tissue regardless of sex and race/ethnicity. The present study indicates that fat distribution is more important to metabolic health than overall body fat, and that global measures of adiposity hold value primarily as a measure of abdominal adiposity. Future research is warranted to further examine why there are racial/ethnic differences in the relationship between visceral adipose tissue and subcutaneous adipose tissue and diabetes. This would serve to better address health disparities among racial/ethnic groups, and how practitioners can estimate visceral and subcutaneous fat based on measurements available in clinical settings to better promote public health.

## Data availability statement

The original contributions presented in the study are included in the article, further inquiries can be directed to the corresponding author.

## Author contributions

FX: Conceptualization, Data curation, Formal analysis, Funding acquisition, Methodology, Visualization, Writing – original draft, Writing – review & editing. JE: Conceptualization, Data curation, Methodology, Writing – original draft, Writing – review & editing. DR: Conceptualization, Methodology, Writing – review & editing. MD: Conceptualization, Methodology, Writing – review & editing. IL: Conceptualization, Methodology, Writing – review & editing. GG: Conceptualization, Methodology, Writing – review & editing.
